# A Decision-Analytic Model to Evaluate Cost-Effectiveness of Regional Implementation of a Mobile Stroke Unit

**DOI:** 10.1212/WNL.0000000000213834

**Published:** 2025-07-08

**Authors:** Peter L. van Hulst, Ruben M. van de Wijdeven, Esmee Venema, Florentina M.E. Pinckaers, Myriam G.M. Hunink, Aad van der Lugt, Diederik W.J. Dippel, Hester F. Lingsma, Daniel Bos, Bob Roozenbeek, for the ICAI Stroke Lab

**Affiliations:** 1 Department of Neurology, Erasmus MC University Medical Center, Rotterdam, the Netherlands;; 2 Department of Radiology and Nuclear Medicine, Erasmus MC University Medical Center, Rotterdam, the Netherlands;; 3 Emergency Department, Erasmus MC University Medical Center, Rotterdam, the Netherlands;; 4 Department of Public Health, Erasmus MC University Medical Center, Rotterdam, the Netherlands;; 5 Department of Radiology and Nuclear Medicine, Maastricht University Medical Center, the Netherlands;; 6 Cardiovascular Research Institute (CARIM), Maastricht University, the Netherlands;; 7 Care and Public Health Research Institute (CAPHRI), Maastricht University, the Netherlands;; 8 Department of Epidemiology, Erasmus MC University Medical Center, Rotterdam, the Netherlands;; 9 Center for Health Decision Sciences, Harvard T.H. Chan School of Public Health, Boston, MA;; 10 Department of Clinical Epidemiology, Harvard T.H. Chan School of Public Health, Boston, MA; and; 11 Department of Neurosciences, KU Leuven, Belgium.

## Abstract

**Background and Objectives:**

Mobile stroke units (MSUs) have the potential to improve functional outcome of ischemic stroke patients, through shortening onset-to-treatment times. Previous cost-effectiveness studies have limited generalizability to nonmetropolitan settings and did not evaluate cost-effectiveness over a lifetime horizon. We aimed to develop a regionally adaptable decision-analytic model, to evaluate cost-effectiveness of MSU implementation and to identify the optimal dispatch scenario.

**Methods:**

We developed a generalizable state-transition microsimulation model with modifiable region-specific parameters and dispatch characteristics to evaluate the lifetime cost-effectiveness from a health care perspective of 1-year MSU implementation. We used the southwest of the Netherlands (1,770,000 inhabitants, 1,592 km^2^, 7 primary stroke centers, 2 thrombectomy-capable stroke centers) as an example. Region-specific input parameters for the model, such as population density, age distribution, and driving times, were obtained at the level of postal codes. We developed a virtual cohort of suspected stroke patients based on age-dependent stroke risks and the number of inhabitants per postal code. We compared the combined dispatch of an MSU and emergency medical services (EMS) with dispatch of EMS alone for patients with onset-to-alarm time <6 hours, living within the catchment area of the MSU. In the base case analysis, the MSU could be dispatched to all postal codes in the study region between 7.00 am and 11.00 pm from a central dispatch site. We assessed the long-term cost-effectiveness through incremental net monetary benefits (iNMBs). Discount rates were 1.5% for effects and 4.0% for costs.

**Results:**

In the base case scenario, the MSU was dispatched to 2,080 of 3,628 patients (57.3%) with a suspected stroke and onset-to-alarm time <6 hours, resulting in a lifetime gain of 399 (95% CI 384–414) additional quality-adjusted life years, €3.9 million (95% CI €3.5 million–€4.3 million) cost savings, and an iNMB of €23.9 million (95% CI €22.8 million–€24.9 million). A smaller catchment area for MSU dispatch was associated with increased cost-effectiveness.

**Discussion:**

Adding an MSU to the dispatch strategy for suspected stroke patients is expected to be cost-effective in our region. Our model facilitates evaluation of the cost-effectiveness of MSU implementation in different regions, settings, and scenarios with varying characteristics.

## Introduction

Faster treatment of ischemic stroke leads to better long-term functional outcome, due to strong time-dependent treatment effects of both IV thrombolysis (IVT) and endovascular thrombectomy (EVT).^1-8^ To distinguish between ischemic stroke, hemorrhagic stroke, and stroke mimic, imaging through noncontrast-enhanced CT and CT angiography (CTA) has become instrumental.^9,10^ A mobile stroke unit (MSU) is an advanced ambulance equipped with a CT scanner, a point-of-care laboratory, specialized staff, and telemedicine devices. MSUs facilitate diagnostic workup and treatment of ischemic stroke patients with IVT in the prehospital setting.^11-14^ Acquisition of a CTA scan in the MSU has the additional benefit of discriminating between large vessel occlusion (LVO) and non-LVO ischemic stroke, allowing direct transportation of EVT-eligible patients to thrombectomy-capable stroke centers, eliminating the need for interhospital transfers.^15^

Dispatch of MSU has been demonstrated to decrease the onset-to-treatment time and increase the thrombolysis rate, when compared with dispatch of conventional emergency medical services (EMS), improving the functional outcome after 90 days.^16-18^ Most MSUs are operational in metropolitan areas, and several cost-effectiveness analyses suggest that an MSU might be cost-effective in this setting.^19-21^ However, the generalizability of these studies to other areas is limited because of influential regional characteristics such as population density, hospital distribution, and organization of health care systems. Furthermore, working hours, crew setup, and catchment area vary between studies, causing a variety of possible dispatch scenarios for the MSU.^16,17,19,22,23^ This limits the comparability between studies, and the optimal MSU dispatch scenario remains unclear.

We aimed to develop a decision-analytic model with modifiable region-specific parameters, to evaluate the cost-effectiveness of regional MSU implementation and to identify the optimal dispatch scenario.

## Methods

### Standard Protocol Approvals, Registrations, and Patient Consents

This study was conducted in accordance with recommendations of the Consolidated Health Economic Evaluation Reporting Standards 2022.^24^ According to the Dutch law, this study did not need approval by a medical ethics committee because no individual patient data were used.

### Study Design and Setting

**Figure 1:**
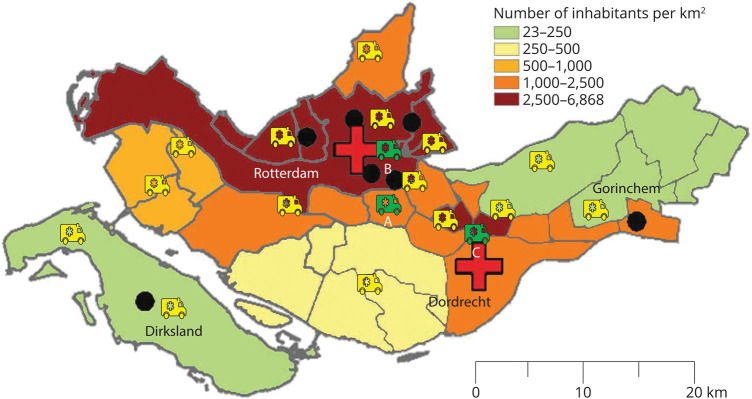
Map of the Study Region With Population Density per Municipality Black dots represent primary stroke centers, and red crosses represent thrombectomy-capable stroke centers. Yellow ambulances indicate regular EMS dispatch sites. Green ambulances indicate regular EMS dispatch sites that could potentially be used as MSU dispatch sites. The letter underneath each green ambulance indicates the location of the MSU dispatch site. A indicates dispatch site Barendrecht, B indicates dispatch site Rotterdam, and C indicates dispatch site Dordrecht. The different colors of the map indicate the number of inhabitants per km^2^ and are obtained per municipality.^46^ EMS = emergency medical services; MSU = mobile stroke unit.

We developed a state-transition microsimulation model to investigate the cost-effectiveness of 1-year regional implementation of an MSU, using the southwest of the Netherlands as an example. This region is defined as the catchment area for ambulance services in the regions Rotterdam-Rijnmond and Zuid-Holland Zuid, encompassing 1.77 million inhabitants, 1,592 km^2^, 7 primary stroke centers, and 2 thrombectomy-capable stroke centers (Figure 1).^25^ We compared a strategy in which an MSU is dispatched in addition to the EMS (MSU + EMS strategy), with a strategy with standard of care (EMS-alone strategy). We evaluated the implementation of an MSU that is staffed with an ambulance driver who is certified to operate the CT scanner and a stroke nurse who is trained to administer IVT treatment to stroke patients. A neurologist and a neuroradiologist are available through telemedicine to supervise neurologic examination, interpret the CT and CTA scan, and decide on treatment initiation. We assumed that there are no differences in diagnostic accuracy between the MSU CT scan and emergency department CT scan. The MSU and EMS are dispatched simultaneously when an emergency call is categorized as a stroke alarm with onset-to-alarm time <6 hours. After initial assessment in the MSU, the patient is transported to the most appropriate hospital (nearest stroke center for the non-LVO patient, nearest thrombectomy-capable stroke center for the patient with LVO) by the EMS. Whenever the MSU is not available (e.g., stroke alarm outside operational hours and MSU already dispatched), only the EMS are dispatched.

### Study Population

Because empirical data on MSU implementation in our study region were not available, we created a virtual cohort of suspected stroke patients per year and per postal code. We obtained the number of inhabitants and age distribution per postal code from Statistics Netherlands (eTable 1)^25^ and data on the incidence and subtype distribution of stroke (TIA, intracerebral hemorrhage [ICH], ischemic stroke) from Dutch Hospital Data.^26^ We obtained the probability of stroke mimic and LVO from the PRESTO study (eTable 2).^27^

### Input Data and Utilities

Quality of life and health care–related costs of suspected stroke patients were evaluated over their remaining lifetime. For patients treated with IVT or EVT, we calculated the treatment effect by modeling the onset-to-treatment time based on individual characteristics (e.g., postal code of residence) and applying a degradation formula, diminishing the effect of treatment per elapsed minute until treatment initiation (eTable 3). This approach was described earlier.^28^ In short, the outcome distributions of untreated ischemic stroke patients with and without an LVO were based on the control groups of previously reported randomized clinical trials (eTable 4).^6,29^ These distributions were shifted for different outcomes based on the estimated treatment effect of IVT and EVT. The beta coefficients of treatment effect and time-dependent decline of treatment effect were estimated with an ordinal logistic regression model using previously reported outcome distributions of treated patients and control patients in different time intervals. The resulting scores on the modified Rankin Scale (mRS) were translated to quality-adjusted life years (QALYs) using utility values published in the literature.^30^

For patients with a stroke mimic, TIA, or ICH, no effect of treatment in the MSU was considered. Therefore, we assumed similar outcomes for these patients in the MSU + EMS strategy and the EMS-alone strategy. Patients with a stroke mimic ended up in a nonstroke health state, and patients with a TIA ended up in mRS score 0 health state. Patients with an ICH transitioned to a health state based on an ICH-specific outcome distribution.^31^

We obtained data on quality of life per mRS category,^30^ death hazard rate ratios per mRS category,^32^ and all cause age-dependent mortality rates^33^ from the literature. We obtained parameters and probabilities on characteristics of (pre)hospital stroke treatment from the PRESTO study^27^ and the publicly available national stroke quality registry, the Dutch Acute Stroke Audit.^34^ Based on expert opinion of 2 neurovascular specialists (D.W.J.D. and B.R.), we assumed that the probability of receiving IVT was 0.55 if patients presented <4.5 hours with an ischemic stroke and the probability of receiving EVT was 0.85 if patients presented <6 hours with an LVO. We modeled onset-to-treatment time based on the individual onset-to-alarm time (eFigure 1),^27^ dispatch-to-arrival time (eFigure 2), time-on-scene,^35^ scene-to-hospital driving time, hospital-specific workflow time (door-to-needle time,^34^ door-to-groin time,^34^ door-in-door-out time^36^), and transfer time. With ArcGIS geographic mapping software, we calculated the dispatch-to-arrival time from the MSU dispatch site and the nearest EMS dispatch site to the postal code of the patient, and we calculated the scene-to-hospital driving time to the nearest primary stroke center and thrombectomy-capable stroke center, respectively. For patients with an LVO who were not evaluated by the MSU and who were first brought to a primary stroke center, an interhospital transfer was needed before EVT could be provided.

### Costs

We assessed the long-term cost-effectiveness of 1-year MSU implementation from a health care perspective. The MSU-related costs consisted of investment costs, operational costs, and personnel costs (eTables 5 and 6). Investment costs are depreciated in 5 years. Acute treatment costs per patient consisted of the cost for MSU and EMS dispatch and, when applicable, cost for IVT and/or EVT treatment. When a patient was treated in the MSU, the emergency department was bypassed and estimated costs were deducted. The rehabilitation costs and costs for long-term care and institutionalization are dependent on the functional outcome after the stroke. The long-term costs per mRS state were derived from the literature and calculated specifically for the Dutch situation (eTable 7).^37^ Costs were valued in Euros and indexed for the year 2021 except for the long-term costs, which were gathered in 2022.

### Data Analyses

**Figure 2:**
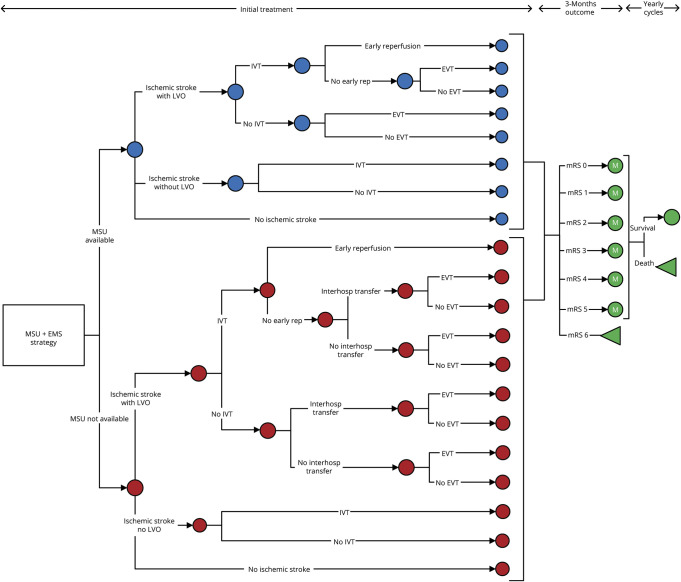
Simplified Version of State-Transition Microsimulation Model In this figure, a simplified version of the MSU + EMS strategy is visualized. Every patient starts as a suspected stroke patient. The MSU will be dispatched if available; otherwise, only the EMS will be dispatched. Every patient will go through the model based on the subtype of stroke and the treatment they receive. Patients will end up in 1 of the 7 health states of the mRS, each with its own quality of life and health care–related costs. The “no ischemic stroke” health state contains patients with stroke mimics, ICH, and TIA. Blue circles represent chance nodes when initial assessment was performed by MSU. Red circles represent chance nodes when initial assessment was performed by EMS alone. The circles marked with an M represent Markov models, and the triangles represent terminal nodes. The EMS-alone strategy is not visualized in this figure, but it has the same structure as the EMS arm in this figure. EMS = emergency medical services; EVT = endovascular thrombectomy; ICH = intracerebral hemorrhage; IVT = IV thrombolysis; LVO = large vessel occlusion; mRS = modified Rankin Scale; MSU = mobile stroke unit.

We developed a state-transition microsimulation model that, owing to its stochastic nature, allows for the simulation of individual patient pathways through various health states over time. By incorporating specific patient characteristics, such as driving time derived from postal codes, our approach provides an estimation of time to treatment for each patient. All patients entered the model in the suspected stroke health state, and they transitioned to 1 of 7 health states of the mRS (0–6) or the nonstroke health state (Figure 2). The health state a patient transitioned to depended on the diagnosis of the patient (stroke mimic, TIA, ICH, ischemic stroke with/without LVO), assigned treatment (IVT and/or EVT), and the onset-to-treatment time (because the effects of IVT and EVT are time-dependent). We assumed that patients remained in the same health state until they died. The first cycle of the model had a duration of 3 months to evaluate 90-day poststroke mRS scores and acute health care costs. The second cycle had a duration of 9 months and covered the remaining part of the first year. Subsequent cycles had a cycle length of 1 year, and half-cycle correction was applied. We ran the model for 100 cycles after which we assumed that all patients had died. We analyzed the model using probabilistic sensitivity analysis with 3,000 iterations to diminish the risk of chance findings and calculated the mean of 10 separate runs of the model.

The discount rate for costs was 4.0% while the discount rate for effects was 1.5%, in accordance with Dutch national guidelines for health economic evaluations.^38^

### Base Case and Alternative Dispatch Scenarios

In the base case analysis, the MSU could be dispatched to all postal codes in the study region between 7.00 am and 11.00 pm from MSU dispatch site A (Barendrecht). To evaluate the most cost-effective dispatch scenario of MSU implementation, we calculated the cost-effectiveness of additional scenarios that consisted of different constellations of the catchment area (based on driving time from dispatch site: entire study region, 25 minutes, 20 minutes, 15 minutes), varying operational hours (12 hours [9.00 am–9.00 pm], 16 hours [7.00 am–11.00 pm], 24 hours per day), and different dispatch sites (A, Barendrecht [suburban]; B, Rotterdam [urban]; C, Dordrecht [suburban]). MSU dispatch sites were selected based on existing infrastructure of EMS. By limiting the catchment area, the MSU will be dispatched only to stroke alarms within a limited driving time from the MSU dispatch site. For every scenario, we calculated the cost-effectiveness and availability of the MSU.

### Outcome Measures

The primary outcome measure was the cost-effectiveness of the implementation of an MSU calculated with the incremental net monetary benefit (iNMB) ([difference in QALY × willingness-to-pay threshold] − difference in cost) using a willingness-to-pay threshold of €50,000 per QALY.^39,40^ The secondary outcome was the difference in onset-to-treatment time between the different assessment strategies.

### Sensitivity Analysis

We created a tornado plot that shows the influence of various model parameters on the outcome of the model, when varied within a prespecified range. The values and ranges for the parameters used in the tornado plot were based on literature and expert opinion (eTable 8).

### Software

The state-transition microsimulation model was developed and analyzed with Amua (version 0.3.0). Driving durations were calculated with ESRI ArcGIS (online, version March 2023). R statistical software (version 4.2.2) and RStudio (version 2022.12.0) were used for the analysis of the availability of an MSU.

### Data Availability

Data not provided in the article may be shared at the request of any qualified investigator for purposes of replicating procedures and results. The model is accessible online on Gitlab at gitlab.com/icai-stroke-lab/MSU-cost-effectiveness-model-Neurology.git.

## Results

### Base Case Scenario

**Figure 3:**
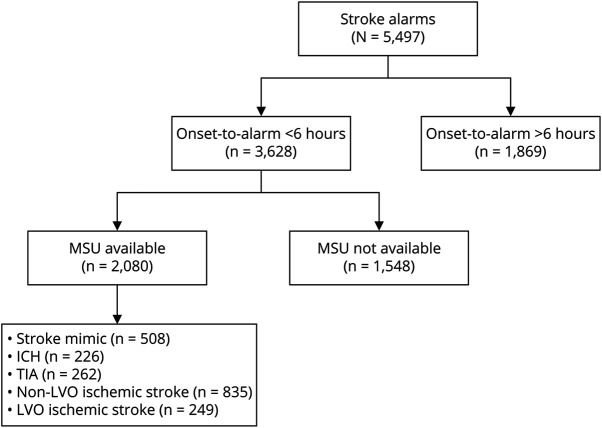
Flowchart of Virtual Cohort This flowchart of the virtual cohort shows the number of patients per subgroup in the virtual cohort. It contains all stroke alarms in 1 year in our study region when the MSU is available for 16 hours per day for all postal codes. Only the patients with onset-to-alarm <6 hours are included in the model. ICH = intracranial hemorrhage; LVO = large vessel occlusion; MSU = mobile stroke unit.

Our study region consisted of 1.77 million inhabitants, with an estimated 5,497 suspected stroke patients per year of whom 3,628 (66%) had an onset-to-alarm time <6 hours. The median age of patients with a suspected stroke was 74 (interquartile range 64–82) years. In the base case, the MSU was available for 2,080 of 3,628 patients (57.3%), resulting in an average of 5.7 dispatches per day. Among the patients who could be treated by an MSU, 996 had no ischemic stroke (i.e., stroke mimic, ICH, or TIA), 835 had a non-LVO ischemic stroke, and 249 had an LVO ischemic stroke (Figure 3). The implementation of an MSU could have saved 189 interhospital transfers for EVT-eligible patients, who would have been transported to a primary stroke center if treated by EMS alone.

**Figure 4:**
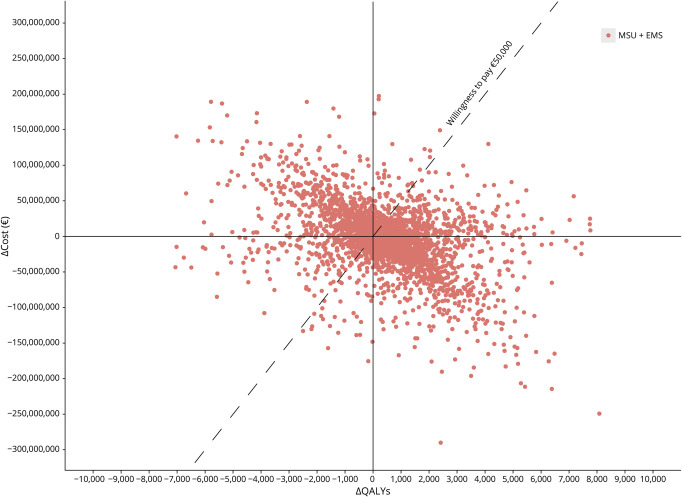
Scatterplot of the Results for Base Case Scenario The scatterplot shows the results for the first out of 10 runs with 3,000 iterations of the probabilistic sensitivity analysis. Every red dot represents the costs and effects of the MSU + EMS strategy compared with those of the EMS-alone strategy (reference strategy). In this scenario, the MSU is dispatched for 16 hours per day to all postal codes in the study region from dispatch site A. The dashed line illustrates the willingness to pay threshold of 50,000 Euros per QALY. On the x-axis is the difference in QALY and on the y-axis is the difference in cost in Euros. EMS = emergency medical services; MSU = mobile stroke unit; QALY = quality-adjusted life year.

Evaluating the lifetime effect of 1-year MSU implementation, the MSU + EMS strategy yielded 399 (95% CI 384–414) additional QALYs, while saving €3.9 million (95% CI €3.5 million–€4.3 million) in costs compared with the EMS-alone strategy (Table 1). This yielded an iNMB of €23.9 million (95% CI €22.8 million–€24.9 million) (Figure 4; eFigure 3). Per suspected stroke patient, 0.11 extra QALYs were gained, corresponding to 40 days in full health. The iNMB became positive within 2 years after the 1-year MSU implementation (eFigure 4).

**Table 1:** Cost-Effectiveness of MSU Implementation From Dispatch Site A

Scenario	Number of dispatches (% MSU available)	Mean gain in effectiveness in QALY (95% CI)	Mean cost-savings in million Euros (95% CI)	iNMB in million Euros (95% CI)
24-h dispatch				
Entire region^a^	2,340 (0.65)	409.2 (362.8–455.6)	3.67 (2.98–4.35)	24.10 (22.90–25.30)
25 min^b^	2,190 (0.67)	442.5 (405.4–479.6)	4.14 (3.96–4.32)	26.30 (25.50–27.00)
20 min^c^	2,007 (0.69)	443.8 (436.2–451.3)	3.94 (3.84–4.04)	26.13 (25.74–26.51)
15 min^d^	1,253 (0.81)	438.2 (432.9–443.5)	3.91 (3.81–4.01)	25.82 (25.53–26.11)
16-h dispatch (7 am–11 pm)				
Entire region (base case)	2,080 (0.57)	399.0 (384.1–413.9)	3.92 (3.52–4.32)	23.90 (22.80–24.90)
25 min	1,953 (0.59)	444.6 (435.3–453.9)	4.02 (3.80–4.24)	26.20 (25.70–26.80)
20 min	1,794 (0.62)	447.5 (440.9–454.1)	4.05 (3.92–4.18)	26.40 (26.00–26.80)
15 min	1,133 (0.73)	436.7 (433.2–440.2)	3.89 (3.78–4.00)	25.70 (25.50–26.00)
12-h dispatch (9 am–9 pm)				
Entire region	1,739 (0.48)	394.8 (373.0–416.6)	3.99 (3.35–4.63)	23.73 (22.13–25.34)
25 min	1,636 (0.50)	435.0 (420.8–449.2)	3.93 (3.73–4.13)	25.68 (24.82–26.54)
20 min	1,507 (0.52)	438.2 (428.4–448.0)	3.92 (3.76–4.08)	25.83 (25.25–26.41)
15 min	959 (0.62)	438.8 (435.4–442.2)	3.89 (3.79–3.99)	25.83 (25.62–26.05)

Abbreviations: EMS = emergency medical services; iNMB = incremental net monetary benefit; MSU = mobile stroke unit; QALY = quality-adjusted life year.

Cost-effectiveness outcomes per scenario for all scenarios from dispatch site A. Gain in effectiveness, cost-savings, and iNMBs for the MSU + EMS strategy are compared with those for the EMS-alone strategy. The base case is the scenario with 16 operational hours per day for the entire region from dispatch site A. The iNMB is calculated with the following formula: iNMB = (difference in QALY × willingness-to-pay threshold) − difference in cost. The willingness-to-pay threshold has a value of 50,000 Euros per QALY.

a Catchment area entire region contains 3,628 suspected stroke patients with onset-alarm time <6 hours.

b Catchment area 25 minutes contains 3,289 suspected stroke patients with onset-alarm time <6 hours.

c Catchment area 20 minutes contains 2,895 suspected stroke patients with onset-alarm time <6 hours.

d Catchment area 15 minutes contains 1,556 suspected stroke patients with onset-alarm time <6 hours.

**Figure 5:**
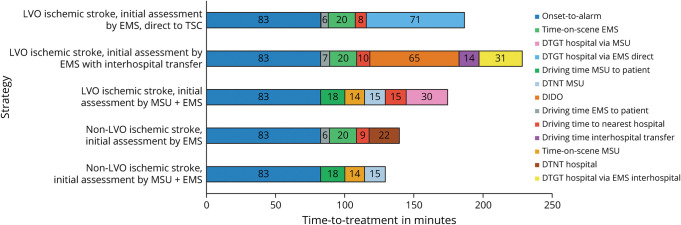
Time Line of Onset-to-Treatment Time and Process Parameters for Base Case Scenario To obtain insights into time-to-treatment for different strategies, we added mean durations per process parameter to create a time line. This figure illustrates the onset-to-treatment time and process parameters for the scenario with 16 operational hours per day for all postal codes from dispatch site A. The strategy called “LVO Ischemic Stroke, Initial Assessment by EMS, direct to TSC” consists of a subgroup of patients, initially assessed by EMS, for whom the nearest hospital is a thrombectomy-capable stroke center. Numbers in the figure illustrate the time spent in minutes per category. DIDO = door-in-door-out time; DTGT = door-to-groin time; DTNT = door-to-needle time; EMS = emergency medical services; LVO = large vessel occlusion; MSU = mobile stroke unit; TSC = thrombectomy-capable stroke center.

The median onset-to-treatment time for non-LVO patients was 9.5 minutes shorter in the MSU than in the EMS. Patients with LVO who would have been transported directly to a thrombectomy-capable stroke center in the EMS strategy were treated 14.5 minutes faster in the MSU strategy. If a patient with LVO had not been transported directly to a thrombectomy-capable stroke center by EMS, treatment by an MSU expedited initiation of EVT by 51.5 minutes (eTables 9–11; Figure 5).

### Alternative Scenarios

**Figure 6:**
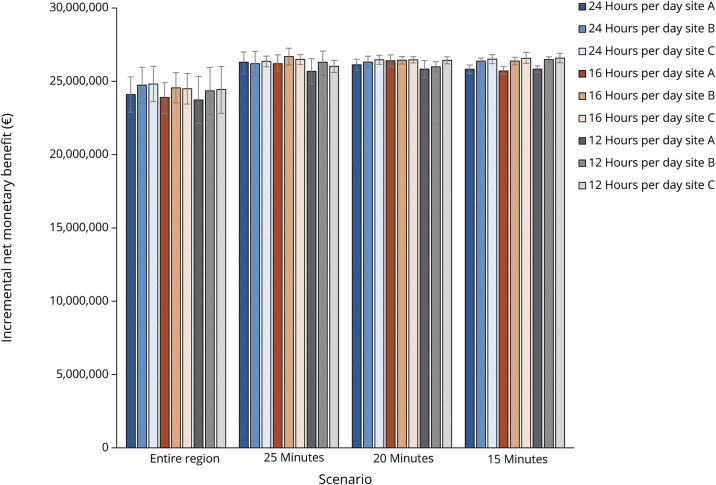
Cost-Effectiveness of MSU Implementation for All Scenarios From All Dispatch Sites This figure illustrates the cost-effectiveness of MSU implementation for all scenarios that were evaluated. Site A indicates dispatch site Barendrecht, site B indicates dispatch site Rotterdam, and site C indicates dispatch site Dordrecht. The scenario in which the MSU is implemented from dispatch site Rotterdam for 16 hours per day within a catchment area of 25 minutes was the scenario with the highest incremental net monetary benefit. The error bars indicate 95% confidence intervals. On the x-axis is the catchment area with maximum driving time in minutes, and on the y axis is the incremental net monetary benefit in Euros. EMS = emergency medical services; MSU = mobile stroke unit.

For different constellations of the catchment area, the number of patients who had an onset-to-alarm time <6 hours was 3,289 (max. 25 minutes), 2,895 (max. 20 minutes), and 1,556 (max. 15 minutes). The onset-to-treatment time was shorter when the catchment area was smaller (eTable 11). In all alternative scenarios, the MSU + EMS strategy yielded more QALYs and lower costs compared with the EMS-alone strategy (Table 1; eTable 12; Figure 6; eFigures 5–7). The scenario with a daily 16-hour deployment in a catchment area of 25 minutes from dispatch site B (Rotterdam) yielded the highest iNMB (€26.7 million).

### Sensitivity Analysis

The probabilities of a stroke mimic, TIA, and ICH had the most significant influence on the effectiveness (eFigure 8), whereas the probabilities of a stroke mimic, TIA, and LVO had the most significant influence on the cost (eFigures 9 and 10). A higher probability of stroke mimics and TIA increased effectiveness and reduced costs because these patients transitioned directly to the mRS score 0 health state with high utilities and low costs. This effect was consistent in both MSU + EMS strategy and EMS-alone strategy. An increase in the probability of LVO resulted in higher cost-effectiveness in the MSU + EMS strategy.

## Discussion

We developed a generalizable decision-analytic model to evaluate the cost-effectiveness of regional MSU implementation. We found that the MSU + EMS strategy yielded greater effects and lower costs, indicating that MSU implementation for patients with a suspected stroke is a cost-effective intervention within our study region. These results are explained by a shorter onset-to-treatment time in the MSU + EMS strategy and, therefore, a better functional outcome and quality of life after the stroke, resulting in a lower necessity for long-term care. The scenario with a daily 16-hour MSU deployment, within a catchment area of 25 minutes from dispatch site B, was the most cost-effective approach of implementing an MSU in our region. Limiting the catchment area seemed to be more efficient compared with coverage of the entire study region. However, the MSU + EMS strategy was more cost-effective in all investigated scenarios, and the differences between scenarios were relatively small. Therefore, other factors such as logistics and crew availability should be considered before implementing an MSU.

Multiple cost-effectiveness analyses, based on empirical MSU data from Melbourne, Berlin, and Norway, showed that MSUs are cost-effective with an incremental cost-effectiveness ratio of AUD30,982 (€18,886), €40,984, and NOK560,020 (€59,524), respectively, per QALY.^20,21,41^ In contrast to our study, none of these cost-effectiveness analyses showed dominance of the MSU + EMS strategy. The differences in cost-effectiveness could be explained by heterogeneity in the organization of health care systems, MSU dispatch strategies, and cost-effectiveness perspective (health care vs societal) and by different approaches in including long-term effects and health care costs in the analyses. It is important to note that the long-term costs per mRS state in our model were higher compared with those in other studies, which had a major influence on the outcome.^21,42^ Therefore, it is necessary to use country-specific estimates of the long-term health care costs when applying the model to other regions.

Existing data on (cost-)effectiveness of MSUs are mainly based on studies in metropolitan areas, limiting its applicability elsewhere. By contrast, the primary strength of our model lies in its broad generalizability, making it suitable for diverse settings. By adjusting the input parameters to the region of interest and by importing data on the regional demographics, driving durations, health care costs, and hospital-specific workflow durations, it is possible to calculate the cost-effectiveness of the implementation of an MSU in other regions.

Another strength of our study is that we investigated the cost-effectiveness over a lifetime horizon to include all effects gained by treatment in an MSU. Most studies evaluated the cost-effectiveness up to 5 years after stroke, underestimating the long-term cost-effectiveness of treatment by an MSU. Moreover, we found that the iNMB becomes positive 2 years after the 1-year implementation, illustrating cost-effectiveness of the MSU + EMS strategy.

In addition, we included all suspected stroke patients, comprising patients with stroke mimic, ICH, and TIA, as well as patients ineligible to receive IVT. Moreover, we used state-transition microsimulation modeling to take individual patient characteristics, such as age and postal code, into account. Therefore, our virtual cohort is representative of a real-world suspected stroke cohort, which helps to envision the expected usage and effect of the MSU in a real-world setting.

Our study has several limitations. Because we do not have empirical data from an MSU in our region, we developed a decision-analytic model in which we had to make estimates and assumptions about the effectiveness and the logistics of an MSU in our study region. We created a virtual cohort with the number and location of suspected stroke patients based on postal codes. These assumptions and estimates influence the accuracy of the outcomes of the model. However, most parameters were obtained from regional data sources, which limits the risk of incorrect assumptions, and we modeled these input parameters with distributions to further account for uncertainty.

We did not model pre-existent morbidity, and we assumed that the patients in our model remained in the same health state until death. Besides, we did not evaluate recurrent stroke or other events affecting mRS scores after a patient ends up in a certain health state. Because this limitation affects both strategies, we expect that the impact on the difference in cost-effectiveness between the strategies will be limited.

We did not evaluate the effect of using prehospital triage instruments in our study. Although such instruments are not implemented in most regions yet, they are promising in optimizing transport strategies for suspected stroke patients. As a result, the difference between the MSU + EMS and EMS-alone strategy may decrease if a triage tool is being used by EMSs.^27,43^

Finally, for patients with ICH, we did not assume an effect of treatment in an MSU. However, the INTERACT 4 study suggests benefit of ultraearly blood pressure management in patients with ICH.^44^ Because the distinction between ICH and ischemic stroke can be made by an MSU, treatment of patients with ICH in a prehospital setting could be improved by MSU dispatch. However, the B_PROUD study did not show benefit and even potentially harmful effects of prehospital blood pressure management in an MSU.^45^ Performing a value-of-information analysis may aid in exploring the potential benefit of conducting more research on providing early treatment for patients with ICH in an MSU.

Given that the model parameters can be adapted to other regions, and because the model has the ability to analyze multiple scenarios, it can be used to calculate the potential cost-effectiveness of an MSU in different regions. An MSU could particularly be helpful in maintaining adequate access to stroke care in rural areas with longer driving durations or lower accessibility to acute reperfusion treatments. Given the limited availability of MSUs, especially in larger catchment areas, it would be interesting to evaluate the cost-effectiveness of implementing more than 1 MSU in the same region. Based on the presented results, the model should be validated and improved using empirical data.

The regional implementation of an MSU is a cost-effective intervention in our study region, and our findings suggest that the MSU + EMS strategy is dominant compared with the EMS-alone strategy. We found that implementing the MSU for 16 hours per day in a catchment area of maximum 25 minutes was the most cost-effective dispatch scenario for an MSU in our region. This model facilitates evaluation of the cost-effectiveness of MSU implementation in different regions, settings, and scenarios with varying characteristics.

## Appendix Coinvestigators

**Table:**

Name	Location	Role	Contribution
Sandra Cornelissen	Department of Radiology & Nuclear Medicine, Erasmus MC, Rotterdam, the Netherlands	Interventional radiologist, ICAI Stroke Lab Study Group member	Discussion and exchange of ideas during weekly meetings
Govert van der Gun	Department of Rehabilitation, Erasmus MC, Rotterdam, the Netherlands	PhD candidate, ICAI Stroke Lab Study Group member	Discussion and exchange of ideas during weekly meetings
Lisa Hoogendam	Department of Plastic, Reconstructive, and Hand Surgery, Erasmus MC, Rotterdam, the Netherlands	Post-doctoral researcher, ICAI Stroke Lab Study Group member	Discussion and exchange of ideas during weekly meetings
Ellen Hu	Erasmus School of Health Policy & Management, Erasmus University, Rotterdam, the Netherlands	PhD candidate, ICAI Stroke Lab Study Group member	Discussion and exchange of ideas during weekly meetings
Xi Li	Department of Public Health, Neurology, Radiology & Nuclear Medicine, Erasmus MC, Rotterdam, the Netherlands	PhD candidate, ICAI Stroke Lab Study Group member	Discussion and exchange of ideas during weekly meetings
Frank te Nijenhuis	Department of Radiology & Nuclear Medicine, Erasmus MC, Rotterdam, the Netherlands	PhD candidate, ICAI Stroke Lab Study Group member	Discussion and exchange of ideas during weekly meetings
Danny Ruijters	Philips Healthcare, Best, the Netherlands	Professor, ICAI Stroke Lab Study Group member	Discussion and exchange of ideas during weekly meetings
Ruud Selles	Department of Plastic, Reconstructive, and Hand Surgery, Department of Rehabilitation, Erasmus MC, Rotterdam, the Netherlands	Professor, ICAI Stroke Lab Study Group member	Discussion and exchange of ideas during weekly meetings
Milou Silkens	Erasmus School of Health Policy & Management, Erasmus University, Rotterdam, the Netherlands	Post-doctoral researcher, ICAI Stroke Lab Study Group member	Discussion and exchange of ideas during weekly meetings
Ruisheng Su	Department of Biomedical Engineering, Eindhoven University of Technology, the Netherlands	Assistant professor, ICAI Stroke Lab Study Group member	Discussion and exchange of ideas during weekly meetings
Sandra Sülz	Erasmus School of Health Policy & Management, Erasmus University, Rotterdam, the Netherlands	Assistant professor, ICAI Stroke Lab Study Group member	Discussion and exchange of ideas during weekly meetings
Theo van Walsum	Department of Radiology & Nuclear Medicine, Erasmus MC, Rotterdam, the Netherlands	Associate professor, ICAI Stroke Lab Study Group member	Discussion and exchange of ideas during weekly meetings
Hans Jansen	Ambulance Service Zuid-Holland Zuid, the Netherlands	Director emergency medical services organization	Advice on design of MSU concept
Arie Wijten	Ambulance Service Rotterdam-Rijnmond, the Netherlands	Director emergency medical services organization	Advice on design of MSU concept
Henk Kerkhoff	Albert Schweitzer Ziekenhuis, Dordrecht, the Netherlands	Neurologist	Advice on design of MSU concept
Pieter Jan van Doormaal	Erasmus MC, Rotterdam, the Netherlands	Interventional radiologist	Advice on design of MSU concept
Marcel Dijkshoorn	Erasmus MC, Rotterdam, the Netherlands	Research laborant CT scan	Advice on design of MSU concept

## Glossary

CTA: CT angiography
EMS: emergency medical services
EVT: endovascular thrombectomy
ICH: intracerebral hemorrhage
iNMB: incremental net monetary benefit
IVT: IV thrombolysis
LVO: large vessel occlusion
mRS: modified Rankin Scale
MSU: mobile stroke unit
QALY: quality-adjusted life year
